# Prevalence of Non-alcoholic Fatty Liver Disease and Its Related Factors in Iran

**Published:** 2016-08-01

**Authors:** I. Moghaddasifar, K. B. Lankarani, M. Moosazadeh, M. Afshari, A. Ghaemi, M. Aliramezany, R. Afsar Gharebagh, M. Malary

**Affiliations:** 1 *Research Committee, Mazandaran University of Medical Sciences, Sari, Iran*; 2 *Health Policy Research Center, Shiraz University of Medical Sciences, Shiraz, Iran*; 3 *Health Sciences Research Center, Faculty of Health, Mazandaran University of Medical Sciences, Sari, Iran*; 4 *Department of Community Medicine, Zabol University of Medical Sciences, Zabol, Iran*; 5 *Department of Basic Science and Nutrition, Health Sciences Research Center, Faculty of Health, Mazandaran University of Medical Sciences, Sari, Iran*; 6 *Research Center for Health Services Management, Institute of Futures Studies in Health, Kerman University of Medical Sciences, Kerman, Iran*; 7 *Urumia University of Medical Sciences, Urumia, Iran*; 8 *Student Research Committee, Mazandaran University of Medical Sciences, Sari, Iran*

**Keywords:** Prevalence, Non-alcoholic fatty liver disease, Meta-analysis [Publication type], Review [Publication type], Iran

## Abstract

**Background:**

Non-alcoholic fatty liver disease (NAFLD) is the most prevalent chronic liver disease in developing and developed countries. Estimating the total prevalence of NAFLD by means of appropriate statistical methods can provide reliable evidence for health policy makers.

**Objective:**

To determine the prevalence of NAFLD in Iran using a systematic review and meta-analysis.

**Methods:**

We identified relevant studies by searching national and international databases. Standard error of the prevalence reported in each study was calculated assuming a binomial distribution. The heterogeneity between the results of the studies was determined using Cochran’s Q and I square indices. We used a random effect model to combine the prevalence rates reported in the studies.

**Results:**

We entered 23 eligible studies in this systematic review investigated NAFLD among 25,865 Iranian people. The total prevalence of NAFLD, prevalence of mild, moderate and severe fatty liver disease were estimated at 33.9% (95% CI 26.4%–41.5%), 26.7% (95% CI 21.7%–31.7%), 7.6% (95% CI 5.7%–9.4%), and 0.5% (95% CI 0.1%–0.9%), respectively. The majority of studies reported that NAFLD was more common among men (seven of eight studies), obese person (15 of 15 studies), older people (10 of 10 studies), patients with systolic hypertension (5 of 8 studies), patients with diastolic hypertension (7 of 9 studies), patients with hypertriglyceridemia (14 of 16 studies), patients with high HOMA level (4 of 4 studies), patients with metabolic syndrome (4 of 4 studies), and those with elevated serum ALT (8 of 12 studies).

**Conclusion:**

Our study showed that the prevalence of NAFLD in Iran was relatively high and male gender, old age, diabetes, metabolic syndrome, systolic/diastolic hypertension, high serum ALT, and hypertriglyceridemia may be determinants of NAFLD.

## INTRODUCTION

Non-alcoholic fatty liver disease (NAFLD) is the most common ailment among chronic liver disorders in developed and developing countries [1, 2]. Most of the patients are asymptomatic, while some of them may present with fatigue, dyspepsia, right upper quadrant pain, and hepatosplenomegaly [3]. It has been estimated that NAFLD together with the epidemic of obesity, will be a major cause of liver-associated morbidity and mortality by 2030 [4]. 

According to the results of previous studies in different parts of the world, prevalence of NAFLD is 36.8% in Mediterranean region, 5%–24% in China), 20%–40% in Europe, 9%–30% in Japan, 16%–32% in Indian urban areas, and 9% in Indian rural areas. The least prevalence rate in Asian countries is 5% in Singapore [5, 6].

Prevalence of NAFLD is related to several factors such as age, gender, ethnicity, presence of sleep apnea and endocrine system disorders (*e.g.*, hypothyroidism, hypopituitarism, hypogonadism, and polycystic ovarian syndrome) [3]. It is also strongly associated with obesity, insulin resistance, type 2 diabetes mellitus, and metabolic syndrome [7]. Prevalence of NAFLD increases with age and is more common among males aged 45–65 years. It is also increasing rapidly among children together with obesity epidemics [2]. In one study, the prevalence of NAFLD among obese people is reported as 80%, while only 16% of people with a normal body mass index (BMI) without any metabolic risk factors are suffering from NAFLD [3]. In addition, more than two-third of patients with type 2 diabetes have NAFLD [7]. 

Several primary studies have reported different prevalence rates of NAFLD in Iran. Such variation in results makes them inappropriate for policymaking. It is necessary to combine the results of different studies by means of reliable methods [8, 9]. We therefore conducted this study to determine the total prevalence of NAFLD in Iran using a systematic review and meta-analysis.

## MATERIALS AND METHODS

Search Strategy

To find relevant studies published from January 2000 to April 2015, we searched national (*SID, Iranmedex, Magiran, *and* Irandoc*) and international (*PubMed, Google Scholar, Scopus, *and* Science Direct*) databases using the following keywords and their Persian language equivalents: “prevalence”, “NAFLD”, “fatty liver disease”, “hepatic steatosis”, “liver biopsy”, “NASH”, “ultrasonography”, “histopathology”, “fibrosis”, “liver”, “steatosis hepatitis”, “non-alcoholic”, “sonography”, “Iran”, “IR Iran”, “Persia”, “Persian”, “Iranian”. Boolean operators were used appropriately to manage the search results.

The search was conducted independently by two researchers. We also investigated all references to enhance the search sensitivity. One of the research team members randomly evaluated the results of the search and reported that all relevant studies had been identified. Non-electronic articles were also investigated. To find un-published studies, some relevant research centers as well as experts in the field of our study were interviewed.

Study Selection

We extracted full texts or abstracts of all papers identified during the advanced search. After excluding duplicates, irrelevant studies were omitted reviewing titles, abstracts and full texts, respectively. We also investigated all results in order to remove repeated studies and minimize the probability of reprint bias. 

Quality Assessment

Quality of the selected studies was assessed by a previously applied checklist [10]. This checklist used contents of STROBE checklist [11] including 12 questions regarding different aspects of methodology such as type and design of the study, sample size and sampling methods, study population, data collection methods and tools, variables definition, statistical tests, study objectives and presentation of the results according to the objectives. One point was assigned for positive response to each question. Every study with a total sum score of eight or more [10] was considered for the meta-analysis. 

Data Extraction

All the required information such as title; first author name; date and place of the study conduction; type of the study; sample size estimation and patient selection; language of the article; diagnostic criteria used for NAFLD (ultrasonography or liver biopsy); prevalence of mild, moderate, and severe fatty liver; association of NAFLD with factors such as patient’s sex, age, BMI, systolic and diastolic blood pressure, serum AST, ALT, ALP, TG, FBS, HOMA, LDL, HDL, and total cholesterol; and presence of metabolic syndrome were extracted.

Inclusion Criteria

All studies written in Persian and English with the minimum acceptable quality score and reported sample size, as well as total prevalence of NAFLD (mild, moderate, and severe) were included in the current study.

Exclusion Criteria

Studies that did not report the prevalence of NAFLD or sample size, congress abstracts without full text, case-control studies, clinical trials, and case reports where estimating the prevalence was not possible, and studies with quality scores lower than the accepted minimum value did not further analyzed.

Statistical Analysis

Data analyses were performed using STATA SE ver 11 software. Standard error of the prevalence in each study was calculated assuming binomial distribution of the data. Based on the degree of heterogeneity between the results of the studies (Cochran test and I^2^ index), fixed or random effect model was applied to estimate the pooled prevalence of mild, moderate, and severe NAFLD in Iran. In addition, to minimize the random variation between the point estimates of the study results, all results were adjusted using Bayesian analysis. Moreover, sensitivity analysis was used to detect studies influencing the heterogeneity. The point prevalence, weight of each study and 95% confidence interval of the prevalence were illustrated by separate points, sizes of the boxes and crossed lines, respectively. 

## RESULTS

**Figure 1:**
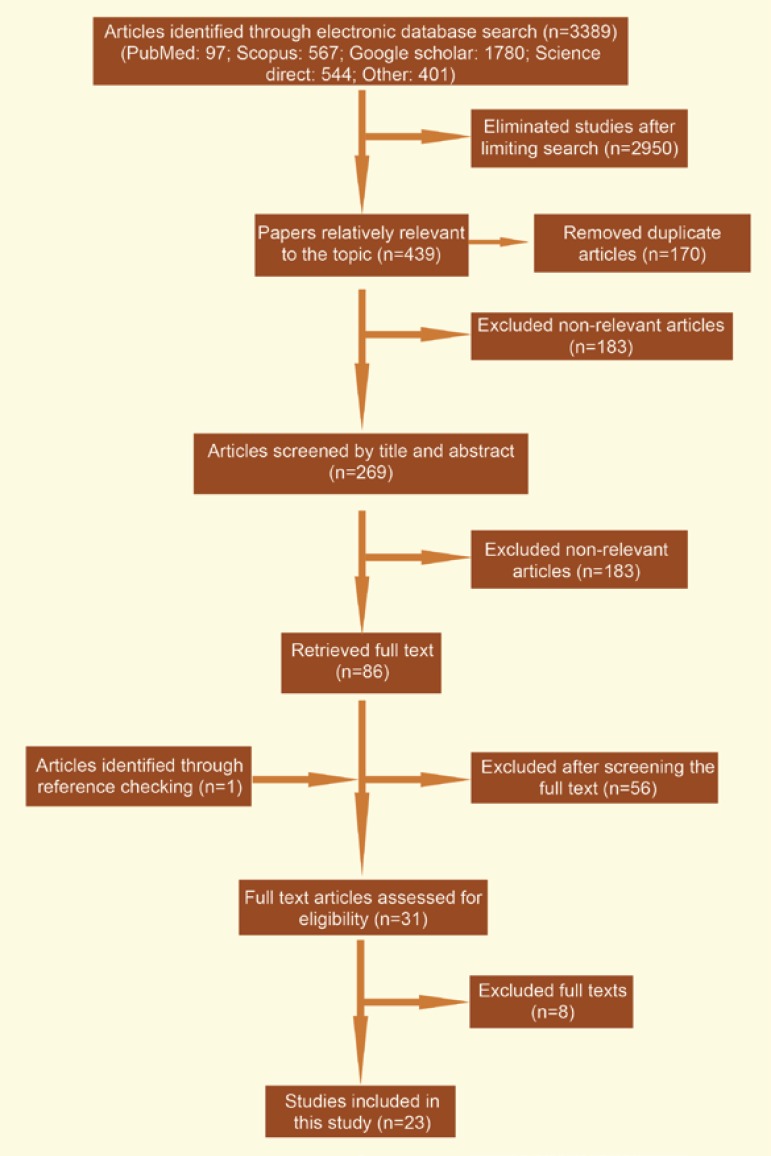
Literature search and review flowchart for selection of primary studies

During our primary search in national and international databases, 3389 articles were retrieved. Restricting the search strategy together with excluding duplicates, 269 articles were remained. Screening via titles and abstracts, 183 papers were found irrelevant. Full texts of the rest of articles were investigated; 56 more papers were found irrelevant. After reviewing references, one article was added to the systematic review. Eight articles were removed after quality assessment and 23 studies [12-34], were finally found eligible for systematic review and the meta-analysis (Fig 1).

All articles were published during 2003 to 2015. Of them, 65% were published after 2010. The study design in all of the studies was cross-sectional. Sampling methods were random (10 studies), facilitated (10 studies), or unknown (three studies). Studies were written in English (19 articles) or Persian (four articles). Study populations were general children and young people (five studies), obese children and young people (four studies), general adult population (11 studies) and diabetic patients (three studies). NAFLD had been diagnosed by ultrasonography (21 studies) or liver biopsy (two studies) (Table 1).

**Table 1:** Baseline characteristics of the included studies in the meta-analysis

ID	First author	Publication year	Study population	Mean age (yrs)	Age range (yrs)	Sample size	Prevalence of NAFLD (%)			
							Total	Mild	Moderate	Severe
1	Alavian	2008	Children and adolescent	—	7–18	966	7.1	6	1.03	0.1
2	Amirkhalili	2014	Adults	45.3	18–90	5023	43.8	—	—	—
3	Eshraghian	2013	Adult	—	>18	832	15.3	—	—	—
4	Hosseinpanah	2007	type 2 diabetes patient	60	41–83	76	82.9	36.8	35.5	10.5
5	Jafarian	2014	Adults	33	20–60	116	12.9	10.3	1.7	0.9
6	Jamali	2008	Adults		18–75	2049	2.04	1.4	0.7	0
7	Bagheri Lankarani	2013	Adults	43.1	18–88	819	43.5	22	16.4	5.1
8	Karimi-Sari	2015	Adults	33.9	—	114	16.7	7.9	3.5	5.3
9	Merat	2009	Type 2 diabetes patient	—	—	172	55.8	—	—	—
10	Merat	2012	Adults	47	11–94	896	16.3	—	—	—
11	Razavizade	2012	Adults	41.6	18–77	254	78.4	58.7	18.5	1.2
12	Montazerifar	2014	Obese adolescents	16	11–19	34	44.1	41.2	2.9	0
13	Rafeey	2009	Children and adolescent	—	6–15	1500	2.3	1.2	0.9	0.2
14	Saki	2014	Obese Children	10.6	5–17	102	54.9	43.1	11.8	0
15	Hosseini	2011	Children and adolescent	12.5	6–18	931	16.8	—	—	—
16	Shahbazian	2011	Type 2 diabetic patients	51.2	—	272	70	42.3	24.3	2.9
17	Taghavi Ardakani	2015	Obese children	9.1	5–15	200	59	54	5	0
18	Tazhibi	2010	Children and adolescent	—	6–18	1107	16.9	—	—	—
19	Adibi	2009	Children and adolescent	12.5	6–18	952	16.9	—	—	—
20	Alavian	2008	Adults	41.6	7–79	1120	9.5	—	—	—
21	Savadkoohi	2003	Adults	43.6	30–60	247	32.8	19.8	12.9	0
22	Arani	2015	Obese children and adolescent	9.5	4–18	360	55.3	49.3	3.9	0
23	Ostovaneh	2015	Adults	—	18–70	7723	35.2	—	—	—

The prevalence of fatty liver varied between 2.04%, in a study conducted by Jamali (2008) on 2049 18–75-year-old people selected from general population, and 82.9%, in a study carried out by Hosseinpanah (2007) on 76 patients with diabetes aged 41–83 years. Having adjusted by Bayesian analysis, the corresponding prevalence rates changed to 2.05% and 80.3%, respectively (Table 2).

**Table 2:** Risk factors related with NAFLD prevalence in Iran by primary studied included to the present study

ID	First author	Sex	Age	BMI	BPD	BPS	AST	ALT	ALP	TG	FBS	HOMA	LDL	HDL	TC	Mets
1	Alavian	NS	<0.001	<0.001	<0.001	<0.001	NS	<0.001	—	<0.001	NS	<0.005	<0.001	NS	<0.001	—
2	Amirkhalili	<0.001	<0.001	<0.05	<0.05	<0.05	<0.05	<0.05	—	<0.05	<0.05	<0.05	—	<0.05	<0.05	<0.005
3	Eshraghian		<0.001	<0.001	<0.001	<0.001	NS	0.001	—	NS	—	—	—	NS	NS	<0.001
4	Hosseinpanah	NS	<0.05	<0.001	NS	NS	NS	NS	NS	<0.05	NS		NS	NS	NS	—
5	Jafarian	<0.05	NS	0.05	—	—	—	—	—	—	—	—	—	—	—	—
6	Jamali	0.005	0.001	0.001	—	—	—	—	—	—	—	—	—	—	—	—
7	Bagheri Lankarani	0.004	<0.001	<0.001	<0.001	<0.001	—	—	—	<0.001	<0.001	—	NS	NS	<0.03	<0.001
8	Karimi-Sari	NS	NS	NS	NS	0.02	<0.001	<0.001	<0.001	0.008	NS	—	NS	NS	NS	—
9	Merat	NS	NS	0.002	—	—	NS	NS	0.01	0.04	NS	—	NS	NS	NS	—
10	Merat	—	—	—	—	—	—	—	—	—	—	—	—	—	—	—
11	Razavizade	NS	NS	NS	—	—	NS	0.005	NS	0.006	—	—	0.006	—	NS	—
12	Montazerifar	—	—	—	—	—	NS	NS	—	NS	NS	—	NS	0.04	NS	—
13	Rafeey	NS	—	—	—	—	—	—	—	—	—	—	—	—	—	—
14	Saki	—	0.001	0.002	NS	0.04	—	0.02	0.04	0.001	0.002	<0.001	NS	NS	NS	—
15	Hosseini	0.04	<0.001	<0.001	—	—	—	—	—	0.01	—	—	—	—	—	—
16	Shahbazian	NS	NS	<0.001	—	NS	NS	NS	NS	<0.001	—	—	NS	NS	—	—
17	Taghavi Ardakani	0.03	0.001	<0.001	—	—	0.001	<0.001	NS	<0.001	NS	—	<0.001	<0.001	<0.001	—
18	Tazhibi	—	—	0.01	—	—	—	—	—	—	—	—	—	—	—	—
19	Adibi	NS	—	<0.001	—	—	—	—	—	—	—	—	—	—	—	—
20	Alavian	<0.001	NS	—	—	—	<0.001	<0.001	NS	0.002	NS	—	—	—	NS	—
21	Savadkoohi	<0.001	NS	—	—	—	—	—	—	<0.001	—	—	—	—	<0.001	—
22	Arani	—	<0.001	<0.001	<0.001	<0.001	—	—	NS	<0.001	NS	0.001	<0.001	NS	NS	—
23	Ostovaneh	—	—	—	—	—	—	—	—	—	—	—	—	—	—	—

The prevalence of mild fatty liver reported in 14 studies, varied from 1.2% in a study conducted by Rafeey (2009) on 1500 patients to 58.7% in a study carried out by Razavizadeh (2012) on 254 individuals. The minimum and maximum prevalence rates for moderate fatty liver were 0.7% (Jamaly) and 35.5% (Hosseinpanah), respectively. The corresponding figures for severe fatty liver were zero (Jamali, Montazerifar, Arani, Taghavi Ardakani, Saki and Savadkoohi studies) and 10.5% (Hosseinpanah), respectively (Table 1).

In the current meta-analysis, based on a random effect model (Q=8037.9, p<0.0001; I^2^=99.7%), prevalence of NAFLD was estimated among 25,865 subjects as of 33.9 % (95% CI: 26.4%–41.5%). According to the sensitivity analysis, studies conducted by Ostovaneh, Rafeey, Jamali, and Amirkhalily were those causing the inter-studies heterogeneity. Excluding these studies, the prevalence of NAFLD was estimated at 36.7%(95% CI: 28.6%–44.9%; Q=2138.6, p<0.0001; I^2^=99.2%). In addition, the prevalence rates of mild, moderate and severe fatty liver disease were estimated at 26.7% (95% CI: 21.7%–31.7%; Q=1460.6, p<0.0001; I^2^=99.1%), 7.6% (95% CI: 5.7%–9.4%; Q=371.7, p<0.0001; I^2^=96.5%) and 0.5% (95% CI: 0.1%–0.9%; Q=72.7, p<0.0001; I^2^=82.1%), respectively.

Of 23 selected articles for the meta-analysis, 20 considered the age groups including individuals aged <18 (eight studies) and >18 (11 studies). The prevalence rates of fatty liver among these age groups were 29.2% (95% CI: 20.4%–38.05%) and 33.7% (95% CI: 20.7%–46.7%), respectively. The overlapping confidence intervals indicate no significant difference of the prevalence rates between the age groups.

Using meta-regression model, study population group was identified as the main source of heterogeneity. The prevalence of NAFLD was increased by 14.7% per one unit increase in the population coding (adult general population: ‘1,’ children general population: ‘2,’ obese children: ‘3,’ and diabetic population: ‘4’). Although each year increase in the publication age increased the NAFLD prevalence by 1.4%. This association was not statistically significant.

The association between NAFLD and sex was reported in 16 studies, eight of which showed significant relationships (prevalence of fatty liver was higher among men in seven studies). Among 17 studies investigated the association between fatty liver and age, 10 studies reported positive correlation with age.

**Table 3:** Factors causing the highest heterogeneity in the current meta-analysis, identified based on univariate and multivariate meta-regression

Variable	Univariate		Multivariate	
	Coefficient	p Value	Coefficient	p Value
Publication	1.4	0.4	2.2	0.1
Population studied	13.7	0.003	14.7	0.001

Out of 17 studies reported association between body mass index and NAFLD, 15 studies observed that NAFLD prevalence was increased significantly with BMI. Systolic and diastolic blood pressures were reported to be significantly associated with NAFLD in five out of eight and seven out of nine studies, respectively.

According to the results of 11 studies assessed the association between NAFLD and serum AST level, the association was found significant in four studies. ALT and alkaline phosphatase were also significantly correlated with NAFLD according to the results of eight out of 12 and three out of nine studies, respectively. 

The association between NAFLD and serum triglyceride was investigated in 16 studies, 14 of which reported significant results. The corresponding figures for LDL, HDL, and cholesterol were four out of 11 studies, three out of 12 studies, and five out of 14 studies. 

Of 11 studies investigated the association between FBS and NAFLD, three studies reported significant associations. All studies reported the significant effects of HOMA (four studies) and metabolic syndrome (three studies) on NAFLD. 

It should be noted that due to the low number of studies investigated, the correlations between NAFLD and factors such as job, family history, smoking, hemoglobin concentration, total bilirubin, and direct bilirubin, were not investigated in this systematic review.

## DISCUSSION

**Figure 2:**
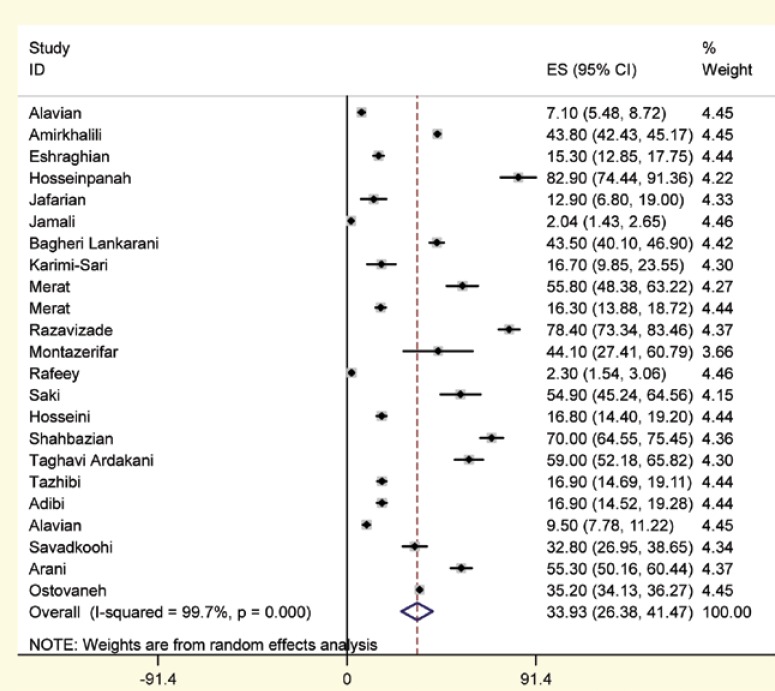
The prevalence of NAFLD among studies included in the meta-analysis and pooled estimate

Our meta-analysis showed a pooled total prevalence of NAFLD of 33.95% (Fig 2). The majority of studies entered in this systematic review, reported that the NAFLD was significantly more common among men (7 of 8 papers), obese people (15 of 15 papers), older ages (10 of 10 papers), patients with systolic hypertension (5 of 8 papers), patients with diastolic hypertension (7 of 9 papers), those with hypertriglyceridemia (14 of 16 papers), high serum HOMA level (4 of 4 papers), patients with metabolic syndrome (4 of 4 papers), and high serum ALT level (8 of 12 papers). Moreover, ultrasonography was the main diagnostic method used for the diagnosis of NAFLD in the studies.

The prevalence of NAFLD among Iranian people has been reported from less than 10% to more than 80% [18, 21]. The prevalence of NAFLD was reported 5%–24% in China, 9%–30% in Japan, 18% in South Korea, 33% in Sri Lanka, 17% in Malaysia, 30% in Indonesia, and 11.5%–42.6% in Taiwan [35]. In a meta-analysis in China the pooled prevalence of NAFLD was estimated at 20.09% [36]. In another study carried out in China, 159 (24.5%) out of 390 healthy people who had undergone ultrasonography, had evidence of fatty liver. These findings were more common among men (27% *vs* 14%) and those with higher BMI. In an Indian survey, among 1168 people investigated with ultrasonography, the prevalence of fatty liver was 16.6%—higher in men (24.6%) than women (13.6%). In that study, age >40 years, male gender, central obesity, BMI >25 kg/m^2^, high FBS level, and high serum ALT and AST, were introduced as risk factors of NAFLD [37].

It has been reported that the prevalence of fatty liver among those living in the territory of Pacific Ocean is 10% so that in some areas, one-third of population have NAFLD. Fatty liver has been reported to be more common among persons with BMI >25 kg/m^2^ compared to those with BMI ≤25 kg/m^2^ in Taiwan (31% *vs* 15%, respectively), Japan (60% *vs* 11%, respectively), and China (39% *vs* 21%, respectively) [38]. According to the results of another study [38], the prevalence of fatty liver among American and European general population was more than 45% which was more common among type 2 diabetic patients (50%), obese people (30%-76%), and those with pathological obesity (>98%).

Based on the results of a study conducted in Malaysia on 399 individuals, the prevalence of NAFLD was 49.6%. According to the multivariate analyses, central obesity (OR=2.20) and elevated serum ALT (OR=1.98) were independent risk factors of NAFLD [39]. Another study carried out on 766 Spaniards aged 17–83 years, the prevalence of NAFLD was 25.8%, significantly (p<0.001) more common among men (33.4%) than women (20.3%). Multivariate analysis showed that male gender (OR=2.34), age (OR=1.04), presence of metabolic syndrome (OR=2.19), insulin resistance (OR=6) and elevated serum ALT (OR=4.21) were risk factors of NAFLD. No association was observed between alcohol use and NAFLD, however, a protective effect of alcohol was found among those without overuse of alcohol (OR=0.93) [40]. 

A study from China showed that 7.27% of 6905 non-obese individuals developed NAFLD. During this cohort, out of 5562 persons without NAFLD at the beginning of the study, 494 (8.88%) developed NAFLD at the end of the follow-up. Moreover, age, gender, BMI, waist circumference, HDL, cholesterol, uric acid, hemoglobin, hematocrit, and platelet count were associated with NAFLD [41].

Xiaona estimated the prevalence of NAFLD among 7152 individuals in Shanghai at 38.17%, which was higher among men increasing with age. In both sexes, prevalence of metabolic factors was higher among those with NAFLD. BMI, waist circumference, weigh:height ratio, blood pressure, blood sugar, total cholesterol, triglyceride, LDL, HDL and uric acid were determinant factors for NAFLD; BMI was the best diagnostic factor for NAFLD. Metabolic factors could increase the risk of NAFLD more than 1.5–3.8 folds [42].

Results of a Brazilian survey conducted on 1280 patients with NAFLD showed that 66.8% of them had hyperlipidemia, 44.7% obesity, 44.4% overweight, 22.7% suffered from diabetes, and 10% were exposed to poisons. Elevated ALT and AST were observed among 55.8% and 42.2% of patients, respectively. Results of liver biopsy among 437 eligible patients (ALT or AST >1.5 times of normal) consisted of steatosis (42%), osteohepatitis (58%), fibrosis (27%), cirrhosis (15.4%), and hepatocellular carcinoma (0.7%) [43].

In Brazil, 2.3% of 1801 students, aged 11–18 years, had NAFLD most of them were white males [44]. Other studies reported a prevalence of 77% (China), 28% (Germany), 42%–53%( Italy), and 74% (USA) [45]. Different prevalence rates of NAFLD among Asian countries have been reported such as 56% (Iran), 35% (Korea), 40%–50% (Japan), and 35% (China). It has also been reported as 74% in North America and 70% in Italy. In Mexico, the prevalence among diabetics was more than that of non-diabetics (18.5% *vs* 7.1%). Another study in India showed that 49% of type 2 diabetic patients had sonographic evidence of fatty liver and prevalence of mild, moderate, or severe NAFLD were 65.5%, 12.5%, and 9.35%, respectively [35]. 

In Saudi Arabia, 47.8% of 230 type 2 diabetic patients studied had NAFLD based on abdominal sonography; no significant difference was observed between men (49.1%) and women (46.3%). The prevalence of NAFLD among 40–59-year-old patients was 52.9% [46]. 

The above-mentioned results showed that NAFLD risk factors among Iranian population are similar to those of other Islamic countries. However, the results of risk assessments in different Iranian studies were not homogenous making it difficult to determine unique risk factors for NAFLD. 

Although liver biopsy is the gold standard for the diagnosis of NAFLD, with a sensitivity of 100% and specificity of 90%, abdominal ultrasonography is the most commonly used method [5, 6]. Another study represented that sonography compared to liver biopsy has 84.8% sensitivity and 93.6% specificity for the diagnosis of moderate to severe fatty liver [2].

The current systematic review and meta-analysis provided reliable estimates of the prevalence of mild, moderate, and severe NAFLD and determined its risk factors. The high degree of heterogeneity among the results of the studies was one of the main limitations of our study. However, according to a meta-regression model, the target population was detected as one of the sources of heterogeneity among primary studies. Moreover, to control this limitation, we applied random effects model to pool the prevalence rates. Therefore, the results should be generalized with caution. Another limitation was inaccessibility to some probable un-published studies. However, we tried to interview with relevant researchers as well as research centers to identify such grey literature. 

In conclusion, our study showed that the prevalence of NAFLD in Iran is relatively high; it is associated with male gender, age, obesity, diabetes, metabolic syndrome, hypertension, raised serum ALT and hypertriglyceridemia. 
